# Scientific crowdsourcing in wildlife research and conservation: Tigers (*Panthera tigris*) as a case study

**DOI:** 10.1371/journal.pbio.2001001

**Published:** 2017-03-22

**Authors:** Özgün Emre Can, Neil D’Cruze, Margaret Balaskas, David W. Macdonald

**Affiliations:** 1 Wildlife Conservation Research Unit, Department of Zoology, University of Oxford, Oxford, United Kingdom; 2 World Animal Protection, London, United Kingdom

## Abstract

With around 3,200 tigers (*Panthera tigris*) left in the wild, the governments of 13 tiger range countries recently declared that there is a need for innovation to aid tiger research and conservation. In response to this call, we created the “Think for Tigers” study to explore whether crowdsourcing has the potential to innovate the way researchers and practitioners monitor tigers in the wild. The study demonstrated that the benefits of crowdsourcing are not restricted only to harnessing the time, labor, and funds from the public but can also be used as a tool to harness creative thinking that can contribute to development of new research tools and approaches. Based on our experience, we make practical recommendations for designing a crowdsourcing initiative as a tool for generating ideas.

## Introduction

The United Nations recently declared that despite the ongoing efforts in conservation, threats to biodiversity will continue to increase and the status of biodiversity will continue to decline [1]. In parallel to the increasing challenges in conservation, there is an increasing emphasis by the research and conservation communities on the need for innovation to overcome these problems (for a broad overview, see references [1] and [2]). Recently, governments of 13 tiger range countries declared that there is a need for “the application of modern and innovative science, standards, and technologies” to increase the effectiveness of efforts to protect tigers in the wild [3]. We responded to this call, inspired by the words of Linus Carl Pauling, the 1954 Nobel Laureate in Chemistry: “The way to get good ideas is to get lots of ideas, and throw the bad ones away.” We designed Think for Tigers, a study to explore whether crowdsourcing (which is an approach to solving problems whose solutions require innovation) could be used to improve the tools used to study and monitor tigers in the wild (see references [4], [5], and [6] for various definitions of crowdsourcing). Crowdsourcing has been used in science, policy, engineering, and the business world with the United States government, NASA, Finnish Parliament, General Electric, and Lego being among the beneficiaries of its use [7–13].

## The need for innovation for protecting tigers in the wild

**Fig 1:**
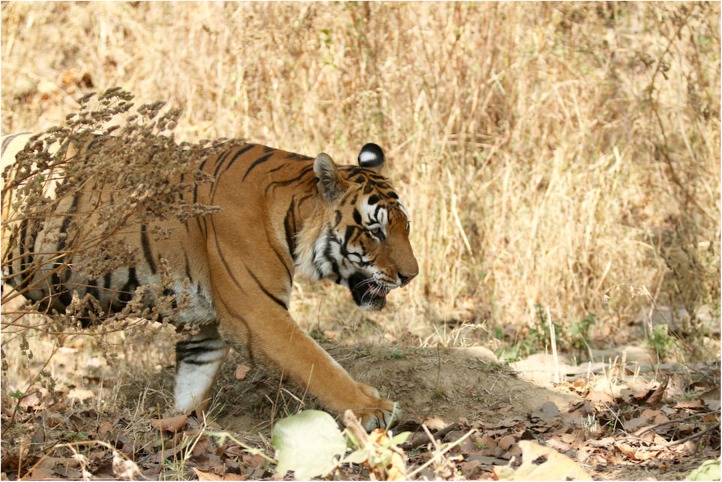
Tigers are 181 kg on average; their lifespan is up to 15 years in the wild and their densities range from 0.7 to 15.84 per 100 km^2^ [15, 16] (Photograph by Özgün Emre Can).

Tigers are in trouble. There are only around 3,200 tigers left in the wild, and their survival is threatened by poaching for the illegal wildlife trade, by habitat loss, and by human–wildlife conflict (Fig 1) [3]. Tiger conservation policy is guided by evidence on their ecology and population dynamics, but the species is elusive, and their study is labor intensive, challenging, and sometimes expensive [14]. Are there better ways forward? We turned to crowdsourcing for an answer.

## What is crowdsourcing?

The term “crowdsourcing” was first coined by Howe [17] and is defined as “the act of taking a job traditionally performed by a designated agent (usually an employee) and outsourcing it to an undefined, generally large group of people in the form of an open call.” There are four main types of crowdsourcing initiatives (Box 1) [18].

**Box 1. Types of crowdsourcing initiatives**

Types of crowdsourcing:

1. Crowd funding: involves an open call to raise money for new projects via an online platform such as Kickstarter.com [18].
2. Crowd labor: involves recruiting of individuals to perform specific tasks such as translation of documents and tagging of digital images of galaxies or camera trap images of animals. Galaxyzoo.org and Snapshotserengeti.org are examples. Other examples are as follows: Folding.stanford.edu is an example where scientists studying Alzheimer, Huntington, Parkinson, and many cancers seek the help of a wide community in running software on participants’ computers. Phlo.cs.mcgill.ca is an initiative intended to solve puzzles confronting genetic disease research. Eyewire.org is a game to map the brain. Proteopedia.org is a wiki-based initiative that aims to collect, organize, and disseminate structural and functional knowledge about RNA, DNA, and other macromolecules. Gene Wiki is a Wikipedia portal that is about applying crowd intelligence to the annotation of gene and protein function.
3. Crowd research: involves asking the public to vote on user-generated ideas, concepts, or products [18]. Voting at the Eurovision song contest is an example.
4. Idea competitions: Involves posting problems online and asking for ideas through online platforms such as Challenge.gov. This represents the idea generation of crowdsourcing [13].

## Who is crowdsourcing for?

Any individual, institution, or community seeking a solution to a problem in hand can pursue a crowdsourcing initiative.

## How to start an idea generation type of crowdsourcing initiative

A crowdsourcing initiative starts with a problem and hopes to end with a solution. In our case, the problem was how to better monitor tigers in the wild. We tackled the challenge in seven steps which, as a guide to others, we summarize here.

### Step 1. Determine the aim of the crowdsourcing initiative and challenge question

Invest considerable time and frame the challenge question [10]. “How might we …” is a phase used by some of the most innovative companies in the world when they tackle the most difficult creative challenges [19]. Framing the question in this way doesn’t imply judgment, helps the crowd to create ideas more freely, and opens up more possibilities [19]. With Think for Tigers, we framed the challenge question as: “How might we better locate, track, and monitor tigers in the wild?” Unsurprisingly, a crucial factor in prompting a useful answer is the selection and phrasing of the challenge question. Questions of a technical bent, perhaps with a narrowly defined remit, may be most tractable to this approach; with hindsight, the breadth of our question may not have stimulated the most productive intensity of technical curiosity.

### Step 2. Determine your target audience

A crowdsourcing initiative should clearly define its “crowd”—i.e., the target audience. Starting with a small community is best [10]. In Think for Tigers, we defined the target audience as anyone over 18 years old and affiliated with a college, institute, or university (as an undergraduate or graduate student, researcher, or academic); with a nongovernmental, governmental, or intergovernmental organization working in the field of nature conservation; or with a technically creative company. Our study was conducted in English.

### Step 3. Determine the idea intake method

**Fig 2:**
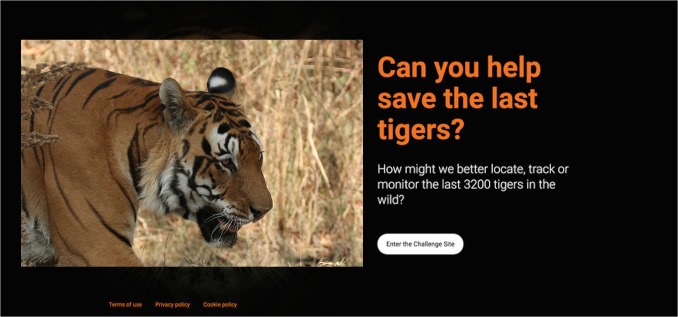
Screenshot of Think for Tigers project home page. The home page was designed to contain three elements—a call for action (Can you help save the last tigers?), the challenge question (How might we better locate, track, and monitor the last 3,200 tigers in the wild?), and a gateway to the challenge portal (Photograph by Özgün Emre Can).

Crowdsourcing activity takes place on a digital platform (i.e., a website) where participants can learn about the challenge and participate. Our website (Fig 2; www.thinkfortigers.org) provided information about the tools researchers and rangers use to study and monitor tigers in the wild. Online crowdsourcing platforms enable participants to register to the crowdsourcing initiative, interact with participants and organizers, and enable organizers to review and manage submissions (such as text, audio, and visual materials submitted by the participants). We used a commercially available crowdsourcing platform to facilitate the submission of ideas from the public and subsequent evaluation by the judges.

Once participants entered the crowdsourcing platform site by clicking the “Enter the Site” button on our home page (Fig 2), they faced ten questions (Box 2) organized in five sections: (1) Idea Intake Method and Standards, (2) Key Benefits, Resources Needed, (3) Implementation, (4) Measuring Success, and (5) Potential Issues or Negative Impact. These questions can be adapted to any idea generation type of crowdsourcing initiative.

**Box 2. Idea intake method and standards**

The Innovative Idea, Product, or Solution

Q1. What is your innovative idea, product, or solution?

Q2. Is your innovative idea, product, or solution about locating or tracking or monitoring tigers? Can it be used for an individual tiger or a group of tigers or both?

Key Benefits

Q3. How does your idea, product, or solution work? How can it be used to locate, track, or monitor an individual or group of tigers in the wild?

Q4. Is your innovative idea, product, or solution a new concept or is it a combination or adaptation of an existing concept? Do you think your idea, product, or solution differs from existing tools or approaches?

Resources Needed

Q5. What materials or technologies are needed to implement your innovative idea, product, or solution?

Q6. What are the financial costs needed to implement your innovative idea, product, or solution? Please try to give a breakdown of major costs per unit/product if applicable.

Q7. How soon do you think your idea, product, or solution could be put into operation?

Implementation and Measuring Success

Q8. How might you measure the effectiveness of your idea, product, or solution?

Q9. What might be the potential obstacles or challenges in implementing your innovative idea, product, or solution? If so, please propose how they might be overcome.

Potential Issues or Negative Impact

Q10. Are there any potential negative impacts that could result from your innovative idea, product, or solution? Please propose how they might be overcome.

### Step 4. Determine how to evaluate the ideas

For the evaluation of entries, best practice involves the prior appointment of a panel of expert judges. The judges should adhere to agreed procedures and criteria for evaluation. We recruited six judges with different expertise (from the fields of carnivore conservation, animal protection, biomechanics, and computer science) and agreed on the following ten criteria to guide their evaluations.

1. Presentation: is the idea clearly described?
2. Solution: does the idea help to locate, track, or monitor tigers?
3. Design: is the idea clearly formulated with sufficient detail?
4. Innovation and uniqueness: is the idea innovative?
5. Technology and materials: are the required technology and materials readily available?
6. Total cost: is the breakdown of major costs presented and feasible?
7. Time required: how quickly can the idea be implemented?
8. Effectiveness: how can the effectiveness of the idea be measured?
9. Obstacles: how might the obstacles that might hinder implementation of the idea be overcome?
10. Negative impacts: how might the negative impacts be overcome, if any?

### Step 5. Determine how to attract people to the challenge

A key factor affecting the success of crowdsourcing initiatives is to reach out to the right audience. Depending on funds available, this can involve a public awareness effort and email and social media tools such as Twitter and Facebook (for helpful tips on how to engage with the public, see reference [20]). We estimated that we were able to reach around 195,000 people via Twitter and around 98,000 people via Facebook during the study period. Further, we contacted a total of 223 people from the world’s top 100 universities as listed in the *Times Higher Education* World University Ranking. We also created a list of nongovernmental organizations (NGOs) working in the field of animal protection and conservation (international as well as major national NGOs in various countries) by searching open sources on the Internet. We contacted 78 people from more than 30 NGOs around the world by email, inviting them to participate in the challenge.

### Step 6. Decide about the incentive and run the challenge

Research shows that it is the interest of individuals and their expectations that primarily drive engagement and participation [21]. We opted to make the award an opportunity [20] and thus offered the winner a ten-day trip to a tiger reserve. Obvious practical considerations include taking account of public holidays and vacation times when setting the start and end dates. We allowed 45 days to carry out the challenge, but longer periods might attract more applicants. However, research showed that the duration of online idea contests had no effect on the average quality of the ideas generated [22]. We received a total of 25 applications from nine different countries (Brazil, Canada, Finland, France, India, Nepal, Netherlands, United Kingdom, and the US) during the 45-day challenge period, with around 1 application per 1.8 days. Most of the participants were among the 301 people who we contacted by email. Ten of the 25 applications received were made by graduate and undergraduate students, 7 applications were made by people based at NGOs, and 4 applications were made by researchers and academics. Four applications were made by people based at governments and technically creative companies.

### Step 7. Identify the winning idea and next steps

**Fig 3:**
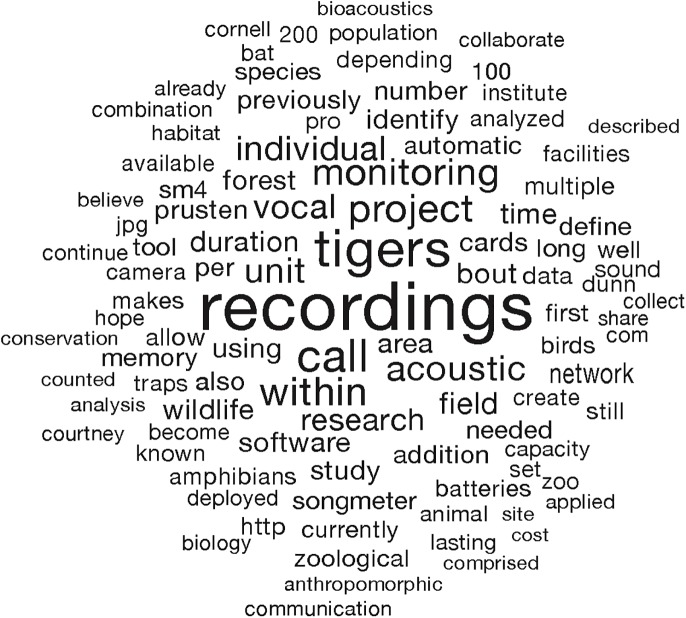
Representation of the winning idea as a “word cloud” based on the words used, length of words, and weighted average of the words.

The ideas submitted to us were diverse, including the following: adapting drones for aerial surveys, cannibalizing old mobile phones for use in specialized camera traps, using trained “sniffer” dogs to track and locate tigers (and poachers), and genetically modifying florescent bacteria to help locate tiger paw prints. The judges selected a winner that combined the fields of bioacoustics, animal behavior, and ecology to study social vocalizations of tigers. The winning idea was to study tigers’ vocalizations with the hope of developing a noninvasive acoustic monitoring for the species (Fig 3) (see “The Prusten Project” [http://www.theprustenproject.org] for more information).

Passive acoustic monitoring has been used to search for the presumably extinct ivory-billed woodpecker (*Campephilus principalis*), rare species such as the little spotted kiwi (*Apteryx owenii*), and endangered blue whales (*Balaenoptera musculus*) as well as many other birds and marine cetaceans [23]. Our team has used acoustic monitoring to measure poaching, as indicated by gunshots [24]. However, the idea needs refinement with respect to technical constraints such as the power requirements of acoustic sensors for long deployments and the challenges of storing data and extracting useful information by analyzing the recordings [23, 25].

## How crowdsourcing and citizen science differ

**Fig 4:**
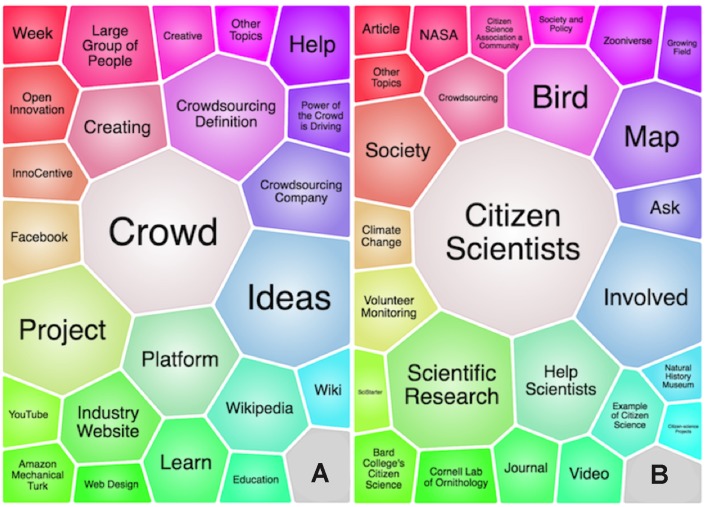
What the Internet “knows” about the terms (A) “crowdsourcing” and (B) “citizen science” based on a cluster search and Lingo clustering algorithm using Carrot^2^ software via 17 Internet search engines [30, 31]. Each cell is a theme created by the algorithm, and the sizes of cells are proportional to the amount of information available in the clustered search results.

Crowdsourcing and citizen science enable professionals and nonprofessionals to voluntarily contribute to science, engineering, and technology. According to the common usage, crowdsourcing differs from citizen science. Citizen science is mostly about distributed data collection and often harnesses the labor of citizens for tedious tasks [26–29], whereas crowdsourcing is about innovation. The cluster search we conducted on the Internet illustrated that crowdsourcing is associated with themes such as “open innovation,” “creative,” “creating,” and “ideas,” whereas citizen science is associated with the themes such as “society,” “scientific research,” and “help scientists” (Fig 4).

## Crowdsourcing: A way to engage people in science

**Fig 5:**
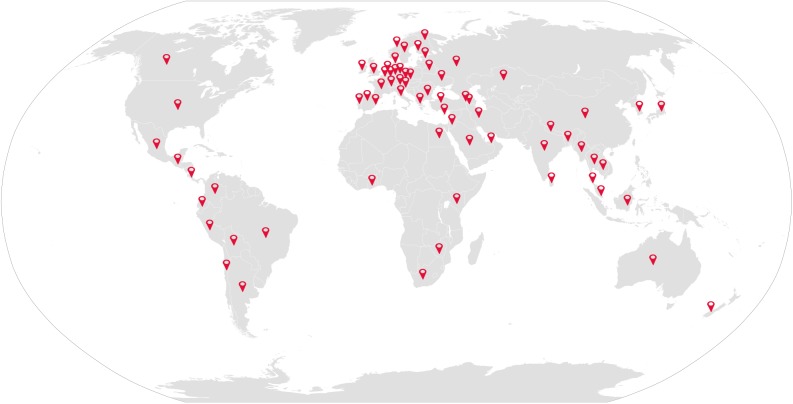
Project’s global outreach. Icons in red indicate the countries from where the project website was visited. Countries and the number of sessions (given in parenthesis) as they are reported by Google Analytics are as follows: Argentina (2), Armenia (1), Australia (32), Azerbaijan (1), Bangladesh (5), Belarus (1), Belgium (8), Belize (1), Bolivia (1), Brazil (97), Bulgaria (4), Cambodia (3), Canada (144), Chile (8), China (59), Colombia (5), Costa Rica (2), Croatia (1), Cyprus (2), Czechia (3), Denmark (18), Ecuador (3), Egypt (1), Estonia (1), Finland (10), France (18), Germany (17), Ghana (1), Gibraltar (1), Greece (11), India (131), Indonesia (42), Iran (2), Ireland (8), Israel (1), Italy (23), Japan (6), Kazakhstan (8), Kenya (2), Lithuania (1), Luxembourg (2), Malaysia (15), Mexico (11), Myanmar (2), Nepal (9), Netherlands (35), New Zealand (26), Norway (4), Peru (6), Poland (2), Portugal (7), Republic of Korea (3), Russian Federation (197), Saudi Arabia (1), Singapore (3), Slovakia (1), Slovenia (1), South Africa (10), Spain (24), Sri Lanka (2), Sweden (5), Switzerland (8), Thailand (4), Turkey (48), Ukraine (7), United Arab Emirates (1), United Kingdom (541), United States of America (350), Zimbabwe (4), and unknown (56).

Crowdsourcing initiatives have the potential to create awareness among both experts and a general public. We used Google Analytics to obtain a snapshot view in which visitor data were not preserved (and so data are anecdotal) to determine details of visits to our project website during the time the challenge was open (between 12 November, 2015 and 28 January, 2016). The website was visited 2,070 times by people from 69 different countries in just 45 days (Fig 5). The study generated the most interest in the UK, US, Russian Federation, Canada, and India. Fifty-four percent of website visitors were male and 46% were female. Most (76.5%) of the website visitors were between 18 and 44 years old. We found Facebook to be more useful in reaching to the public compared to Twitter in terms of effort spent and outreach obtained. Two Facebook posts received 1,633 shares, likes, and comments, reaching a total of 97,999 people. There were around 334 interactions on Twitter, reaching a total of 195,789 people via tweets and retweets.

## Crowdsourcing: An underutilized tool in conservation research

Citizen science enables everyone to contribute to conservation science by helping researchers and practitioners with data collection and conducting time-consuming tasks. Crowdsourcing can be used as a useful tool for innovation but it has not been widely used in the fields of ecology, conservation, and animal protection. While citizen science brings alive the aphorism that many hands make light work, crowdsourcing facilitates many brains making bright ideas.

## Ethics statement

The study was initiated after receiving the relevant research ethics approval (Reference No: R42804; 3 November, 2015) from the Central University Research Ethics Committee of the University of Oxford.
